# Influence of localized roadway surface obstacles on vehicular emissions under real-world urban driving conditions

**DOI:** 10.3389/fdata.2026.1807559

**Published:** 2026-06-23

**Authors:** Victor Cardoso Oliveira, Thiago Iachiley Araújo de Souza, Nicole Souza Batista, Bruno Vieira Bertoncini, Verônica Teixeira Franco Castelo Branco

**Affiliations:** 1 Research Group Transport and Environment (TRAMA-UFC), Department of Transportation Engineering, Federal University of Ceará, Fortaleza, Ceará, Brazil; 2 Federal University of Ceará, Sobral, Ceará, Brazil

**Keywords:** air pollution, climate change mitigation, computer vision, pavement surface irregularities, portable emission measurement system, real-world vehicular emissions, urban transport infrastructure, vehicular exhaust emissions

## Abstract

**Introduction:**

Vehicular emissions are a major source of air pollution in tropical urban environments. While the impacts of technology, traffic flow, and driving behavior on pollutant formation are well established, the influence of pavement surface remains insufficiently understood. Pavement defects such as potholes, cracks, and depressions disturb vehicle operation and may increase real-world emissions. This study evaluates the influence of pavement obstacles on emissions of CO_2_, CO, and NO_x_ across five urban road segments in Fortaleza, Brazil.

**Methods:**

A portable emissions measurement system (PEMS) collected second-by-second exhaust data during real driving. Roadway surface obstacles were captured through windshield-mounted images acquired at 1 Hz. A broader image pool comprising 44,175 roadway images was assembled from multiple urban roads for model development. From this pool, a final annotated dataset comprising 1,812 images with 3,158 labeled obstacles was used for training, validation, and testing the YOLOv8n detector, which was then applied to the five monitored road sections used in the synchronized emission analysis. Emission data and obstacle locations were synchronized, enabling comparison of pollutant rates along obstacle-present and obstacle-free segments, with emphasis on features likely to influence short-term driving behavior. Detection performance was evaluated using precision, recall, and mAP metrics.

**Results:**

Road segments with higher obstacle occurrence presented elevated emission rates. In the full dataset, maximum values reached 478.2 g/km for CO_2_, 491.96 mg/km for CO, and 100.266 mg/km for NOx. A filtered analysis excluding curves, intersection buffers, and visible traffic or pedestrian interference showed that obstacle-present observations still exhibited higher emissions than obstacle-free observations, with average increases of 26% for CO_2_, 31% for NOx, and 42% for CO. Spatial mapping showed that emission hotspots tended to occur in areas with frequent roadway surface obstacles and operational disturbances.

## 1 Introduction

Urban transportation is one of the dominant sources of atmospheric pollutants in rapidly growing cities, contributing significantly to deteriorating air quality, greenhouse gas emissions, and climate-related impacts (World Health Organization, 2022; Intergovernmental Panel on Climate Change, 2022). Exhaust emissions from internal combustion vehicles—particularly carbon dioxide (CO_2_), carbon monoxide (CO), and nitrogen oxides (NO_x_)—are strongly associated with adverse health outcomes and increased radiative forcing, especially in tropical and densely populated urban areas (Molina et al., 2019; Fuller et al., 2022). Understanding the mechanisms that modulate vehicular emissions under real-world conditions is therefore essential for improving urban sustainability strategies and achieving international climate targets (Fiore et al., 2015; EPA, 2024). Because many of the same sources that degrade urban air quality also contribute to climate forcing, transport emission mitigation can generate simultaneous co-benefits for public health and climate policy, which reinforces the relevance of studies focused on real-world emission drivers rather than only certification values (Fiore et al., 2015; Fuller et al., 2022).

While technological, behavioral, and fleet-related factors play essential roles in determining vehicular emissions, road infrastructure has emerged as an additional influential—yet often overlooked—dimension. Pavement characteristics such as surface type, roughness, irregularities, and structural degradation affect vehicle dynamics by altering acceleration, braking frequency, and engine load (Shahare et al., 2017; Azevedo et al., 2021). Earlier modeling frameworks, including HDM-4, acknowledge these relationships but often rely on oversimplified assumptions or lack calibration for tropical urban environments (Chatti and Zaabar, 2012). Such limitations are particularly relevant for Brazil, where climatic stressors, underfunded maintenance systems, and rapid urbanization contribute to accelerated pavement deterioration (Salvo Junior and Souza, 2018). Recent evidence has further shown that road characteristics such as road grade, road surface, traffic state, and rolling resistance can materially influence fuel consumption and CO_2_ emissions in real driving conditions, indicating that infrastructure should be treated not merely as a background condition but as an operational factor affecting energy demand and pollutant formation (Fontaras et al., 2017; Nitoiu et al., 2025; Llopis-Castelló et al., 2026). In addition to these direct mechanisms, pavement roughness may also influence emissions indirectly by affecting drivers' speed choice and short-term operating behavior, suggesting that part of the infrastructure–emission relationship may be behaviorally mediated under real driving conditions (Chen et al., 2024). This point is especially relevant in tropical cities, where high solar radiation and marked rainfall seasonality may accelerate pavement aging and increase the frequency of localized surface irregularities over time (Rocha et al., 2019).

Recent research has reinforced the need to incorporate infrastructure-related variables into emission analyses. Oliveira et al. (2025), for example, used controlled PEMS measurements under nearly constant 60 km/h operation to show that changes in pavement surface type (concrete vs. asphalt) lead to substantial differences in NOx emissions: average NOx levels on Portland cement concrete sections were about 50% lower than on asphalt sections, while CO_2_ and CO emissions remained statistically similar between the two surfaces. Although that study focused on isolating the effect of pavement type, the results suggest that pavement physical properties influence combustion processes and operating regimes, in line with other work relating surface type and thermal behavior to emission characteristics (Azevedo et al., 2021). Similarly, Llopis-Castelló et al. (2026), using a before–after approach based on connected vehicle data, reported that a 27% reduction in pavement roughness after rehabilitation was associated with reductions of approximately 10% in both fuel consumption and CO_2_ emissions, with particularly strong effects on uphill segments. In addition, Nitoiu et al. (2025) emphasized that real urban driving cycles are jointly shaped by road and traffic characteristics, further supporting the argument that infrastructure-related variables should be explicitly considered when examining on-road pollutant emissions. Chen et al. (2024) further advanced this discussion by proposing a carbon-emission calculation method that explicitly considers the relationship among pavement roughness, vehicle carbon emissions, and driving speed, emphasizing that roughness can affect emissions not only through rolling resistance but also through changes in driver speed selection.

However, while pavement type has received some attention in the literature, real-world urban driving occurs on pavements that are often irregular, degraded, and full of localized obstacles—conditions that induce transient dynamics strongly associated with fuel overconsumption and emission spikes. Driving disturbances caused by potholes, cracks, and depressions increase speed variability and engine transients, amplifying emissions in ways that traditional models and laboratory cycles fail to capture (Chen et al., 2024; Muhammad et al., 2024; Shahare et al., 2017). This gap underscores the need for high-resolution, data-driven approaches capable of capturing the relationship between pavement surface obstacles and vehicular emissions. This perspective is consistent with recent behavior-based emission studies showing that real-world pollutant levels are highly sensitive to driving patterns, rapid acceleration, braking events, speed regulation, and spatially concentrated high-emission zones (Muhammad et al., 2024).

In the present study, the analytical focus is not on deriving a comprehensive pavement condition index, but on identifying localized road surface obstacles that are likely to alter driver behavior and consequently, vehicle operating patterns. In this sense, the objective is to detect surface features that may induce steering corrections, braking events, speed adjustments, or transient acceleration responses, since these short-term changes in driving behavior are the mechanisms most directly linked to real-world emission variability. This distinction is important because traditional pavement assessment indices, such as the Pavement Condition Index (PCI), are designed to represent overall pavement condition for engineering and management purposes and may include forms of distress that do not necessarily affect driving behavior or vehicle operation in a measurable way. Therefore, the image-based procedure adopted here should be interpreted as a method for detecting behaviorally relevant roadway obstacles, rather than as a substitute for conventional pavement condition assessment tools.

Real-world emissions frequently diverge from laboratory-based certification values, a gap that can exceed 40% according to large-scale evaluations of European passenger vehicles (Fontaras et al., 2017). Fontaras et al. (2017) demonstrated that certification procedures such as the new european driving cycle (NEDC)—a chassis-dynamometer test designed in the 1980's and characterized by low accelerations, gentle speed transitions, and short dynamic variability—systematically underestimate fuel consumption and CO_2_ emissions due to unrealistic boundary conditions and the omission of influential real-world factors, including road grade, surface roughness, and auxiliary loads. Their review also indicates that the real-world gap may vary substantially depending on traffic conditions, road loads, and external demands, showing that average laboratory representations are insufficient to explain on-road variability (Fontaras et al., 2017). This interpretation is consistent with earlier work demonstrating that real-world speed profiles, facility type, and transient operating modes are critical for emission estimation, especially because instantaneous engine load—not just average speed—strongly affects pollutant generation (Zhai et al., 2008). More recently, telemetric frameworks based on driving behavior and vehicle specific power (VSP) have reinforced that emissions should be interpreted through real-time vehicle operation, since speed, acceleration, road type, and driver behavior can generate spatially and temporally concentrated emission peaks under urban conditions (Muhammad et al., 2024).

Recent technological advances offer promising pathways to address this gap. PEMS allow second-by-second quantification of exhaust gases under real driving conditions (Weiss et al., 2011; EPA, 2024), while modern computer vision algorithms, including YOLO-based architectures, enable automated detection and geolocation of pavement surface obstacles from onboard video (Batista et al., 2025). Integrating both technologies enables a level of spatial and temporal alignment between emission events and road surface conditions that was not previously feasible.

In methodological terms, these data sources are complementary: broader approaches based on connected vehicles or floating car data can improve network representativeness, whereas instrumented-vehicle approaches using PEMS provide the temporal resolution needed to detect short-duration emission peaks associated with localized road disturbances (Weiss et al., 2011; Llopis-Castelló et al., 2026). Similarly, telemetric data-driven approaches have been proposed to capture high-risk driving patterns and high-emission zones under real-world conditions, demonstrating the growing relevance of spatial–temporal and behavior-sensitive methods for urban emission assessment (Muhammad et al., 2024). At the same time, recent reviews in computer vision indicate that deep learning has been increasingly applied to pavement distress detection, classification, segmentation, quantification, and condition-related assessment, while also highlighting persistent challenges related to generalization, image quality, defect heterogeneity, and practical implementation (El Hakea and Fakhr, 2023). Recent YOLOv8-based studies have also shown that road distress detection performance can vary according to roadway class, pavement material, image quality, and visual context, highlighting the importance of preprocessing, dataset diversity, and careful interpretation of detector outputs in practical applications (Li et al., 2024). Within this context, the image-based analysis adopted here is intended to detect visually identifiable road surface obstacles from onboard video, rather than to estimate a comprehensive pavement management metric.

Unlike prior studies focused on pavement type or average roughness under steady-state conditions, this research advances the literature by investigating the relationship between localized roadway surface obstacles and vehicular emissions under real-world urban driving conditions. By focusing on transient emission dynamics associated with discrete, driver-relevant road obstacles, the study integrates high-resolution PEMS measurements with YOLO-based computer vision detection synchronized in space and time. The proposed data-driven framework enables event-level emission analysis and provides new empirical evidence linking localized roadway disturbances to increased pollutant output in tropical urban environments. In this sense, the study contributes not only by combining two high-resolution data streams, but also by explicitly framing obstacle occurrence as an operational proxy of potential driving disturbance.

## 2 Materials and methods

### 2.1 Study area

**Figure 1:**
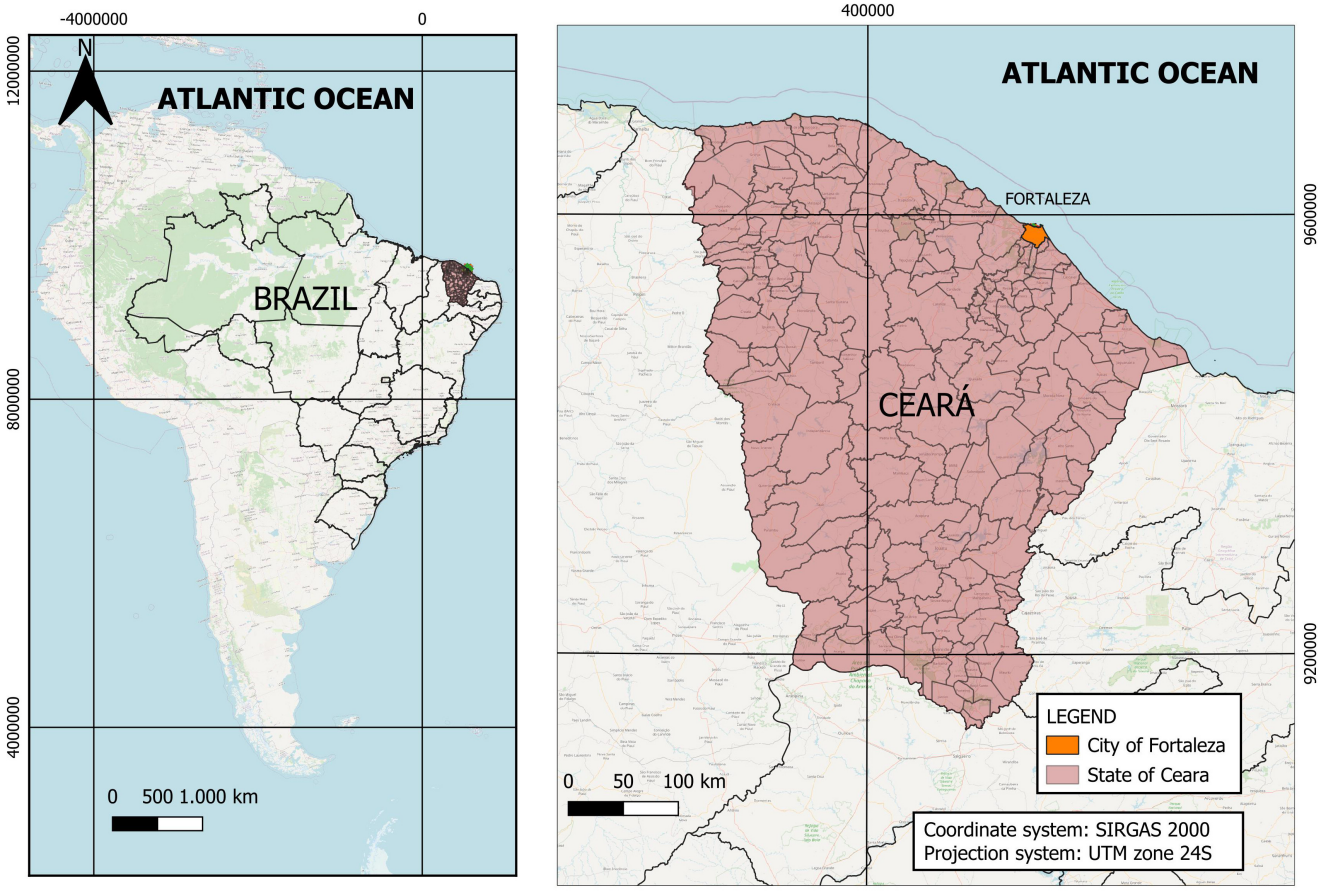
Geographic location of the study area in Brazil.

The study was conducted in the tropical metropolis of Fortaleza, located in the state of Ceará in northeastern Brazil (Figure 1). The region is characterized by consistently high levels of solar radiation, with mean daily global values exceeding 5 kWh·m^−^^2^ as demonstrated by long-term analyses of observational data (Rocha et al., 2019). These conditions, typical of coastal semi-arid environments, contribute to elevated surface temperatures and accelerated oxidative aging of asphalt materials. High heat fluxes combined with ultraviolet exposure promote binder hardening and stiffness increase, phenomena widely associated with premature pavement deterioration. Such climatic conditions are also relevant from an operational perspective, because they contribute to the recurrence of localized surface anomalies that may become visually detectable road obstacles during urban driving.

Fortaleza also exhibits a distinct precipitation regime marked by strong seasonality, with most rainfall concentrated between February and May (Rocha et al., 2019). This pattern results in alternating cycles of moisture infiltration and prolonged dryness throughout the year, further intensifying pavement degradation mechanisms such as raveling, cracking, and surface disintegration. These environmental stressors make the region a representative setting for analyzing real-world interactions between pavement obstacles and vehicular emissions, given that deteriorated pavements are common in tropical metropolitan areas and significantly influence vehicle dynamics. In addition, the combination of climatic exposure, recurrent maintenance needs, and heterogeneous urban traffic conditions makes Fortaleza an appropriate case study for investigating whether visually identifiable road obstacles are associated with short-term changes in pollutant emissions under actual operating conditions.

### 2.2 Road segment selection and characterization

**Figure 2:**
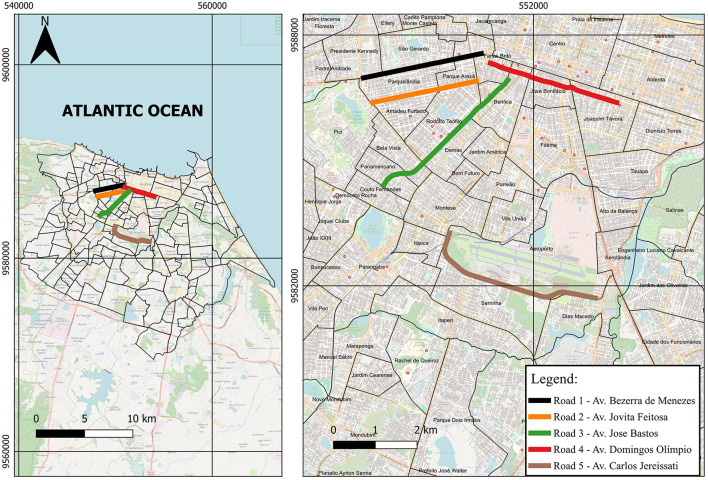
Location of the five urban road sections analyzed in the study.

Five urban road segments were selected based on visual inspection, prior geotechnical assessments, and their representativeness of typical surface conditions found in the city (Figure 2). The selected segments included both well-maintained pavements and sections exhibiting varying degrees of degradation, such as potholes, cracks, depressions, and localized surface obstacles. Each segment also varied in geometric design, including lane width, curvature, longitudinal grade, and posted speed limits, ensuring coverage of heterogeneous operational conditions. These characteristics were documented to contextualize driving dynamics and support subsequent segmentation analyses.

Traffic characteristics were also considered during selection. The study design prioritized segments with moderate flow during non-peak periods to minimize confounding effects associated with congestion or stop-and-go patterns. All segments were surveyed in the outbound and inbound directions to capture directional differences in road condition and operational variability. Additional contextual variables—including surrounding land use, shading conditions, and potential sources of visual occlusion—were observed to ensure consistency and reliability in data collection.

### 2.3 On-board instrumentation

A PEMS was installed in a light-duty passenger vehicle used as the test platform. The PEMS collected second-by-second measurements of CO_2_, CO, and NO_x_, along with vehicle speed, engine load, exhaust flow rate, and GPS coordinates. All sensors were calibrated according to manufacturer requirements before each field session. Calibration procedures included zero-gas checks, span verification, and leak detection to ensure the accuracy of gaseous analyzers and flow measurement systems. The use of a single instrumented vehicle ensured consistency in vehicle response, instrumentation setup, and driver–vehicle interaction across all monitored corridors, although it does not imply fleet-level representativeness.

The PEMS was secured in the cargo area of the test vehicle to avoid vibration-induced interference. Sampling lines were mounted externally following safety guidelines to avoid exhaust backflow and ensure representative pollutant capture. The system operated continuously throughout each run, generating a high-resolution dataset capable of capturing transient changes in emissions associated with pavement-induced variations in vehicle operation. This second-by-second resolution was particularly important because the study focused on short-duration road disturbances that may produce localized and transient emission responses.

**Figure 3:**
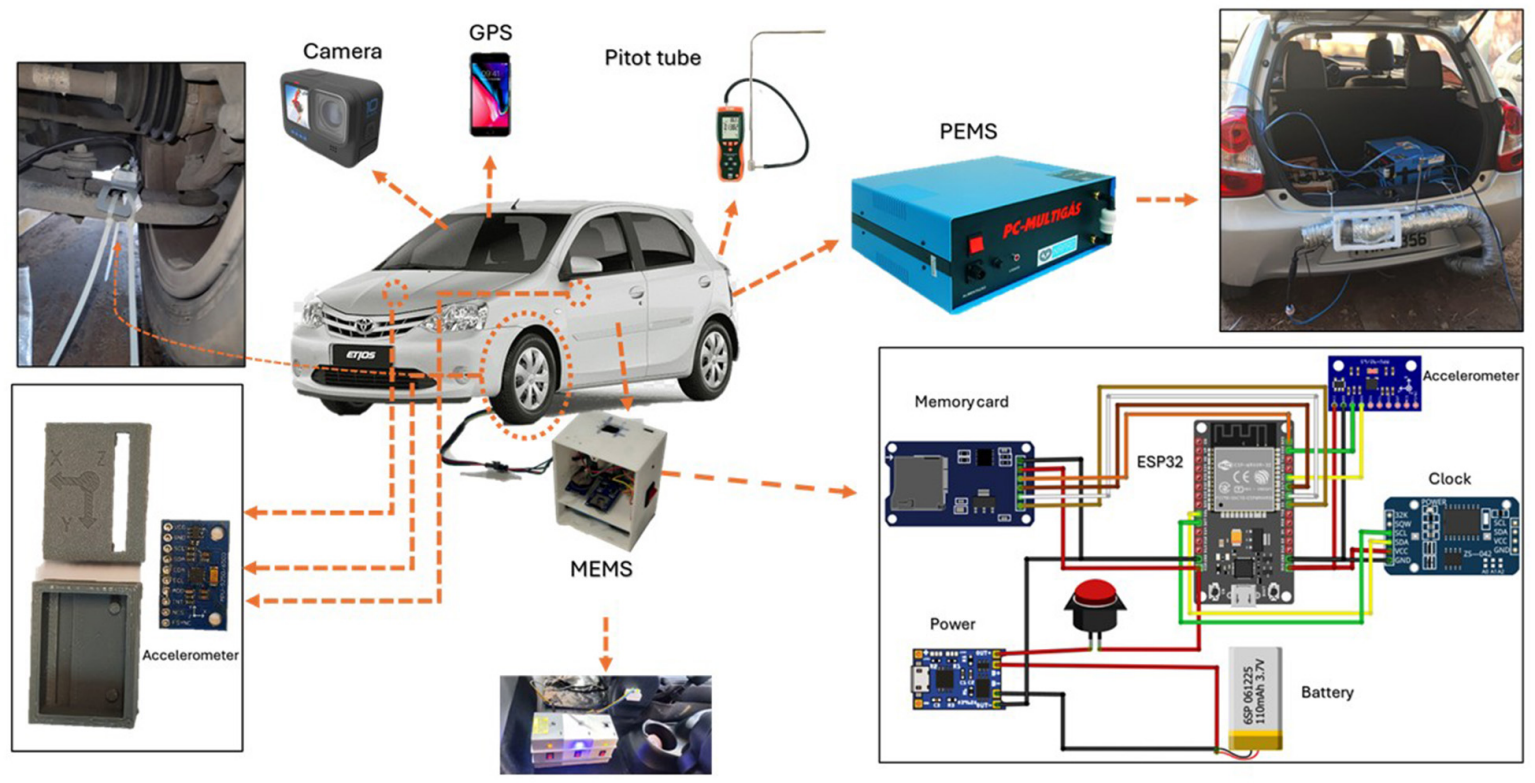
Overview of the integrated data acquisition system used for real-world emission and pavement surface obstacle monitoring.

A GoPro Hero 10 Black camera was mounted on the vehicle windshield to obtain high-definition video of the road surface. The camera recorded at a minimum of 1,080p and 60 frames per second, enabling precise visualization of pavement obstacles. In addition, a micro-electro-mechanical system (MEMS) sensor module was installed inside the vehicle to record inertial data such as acceleration, vibration, and motion dynamics. To maintain consistency, the camera was fixed in the same orientation in all data collection sessions, reducing variability in perspective and ensuring stable image geometry for automated detection. The assembly diagram is represented in Figure 3. For the purposes of the present analysis, the video stream was later sampled at 1-s intervals so that one representative image could be synchronized with each second of PEMS data. Thus, although the original video was continuous, the image-based analysis was intentionally designed around temporally aligned frames rather than around a fixed spatial acquisition interval.

All video files were time-synchronized with PEMS data using internal timestamps and GPS metadata. Environmental conditions, such as lighting, cloud cover, and rainfall, were recorded at the beginning and end of each session to identify potential visual interference. Only recordings obtained during favorable weather and adequate visibility were included in the analysis, ensuring the reliability of subsequent computer vision processing. Because frame extraction was based on a fixed temporal interval rather than a fixed traveled distance, the spatial spacing between consecutive analyzed frames varied with instantaneous vehicle speed. Therefore, the procedure was not intended to generate a gap-free pavement inventory, but rather to identify temporally synchronized road surface obstacles with potential short-term operational relevance for driver behavior and emissions.

### 2.4 Obstacle detection using YOLO

With the aid of artificial intelligence and machine learning, damage detection tasks are increasingly replacing manual operations with more adaptive solutions (Xia et al., 2026). In this sense, the emergence of cutting-edge deep learning technologies has significantly enhanced the scope, intelligence, accuracy, speed, and efficiency of detection algorithms, as is the case with the YOLO family (Yang et al., 2025). Recent papers have shown that computer vision methods are now widely applied to pavement distress detection, classification, segmentation, and condition-related screening, although practical challenges remain regarding image quality, model generalization, distress heterogeneity, and interpretation of the detected features for engineering applications (El Hakea and Fakhr, 2023).

In this study, localized roadway surface obstacles were identified using a computer vision pipeline based on the YOLOv8 architecture. For model development, a broader image pool comprising 44,175 roadway images was assembled from multiple urban roads in Fortaleza, including but not limited to the five road sections selected for the emission analysis. This broader image pool was used to increase the diversity of pavement appearances, lighting conditions, obstacle types, and roadway contexts available for model training and evaluation. The images were extracted from windshield-mounted video records and processed through a standardized workflow. Frames containing visible asphalt pavement were retained, cropped to isolate the road surface, and filtered to remove irrelevant upper-frame content.

The images were then resized to 640 × 640 pixels in accordance with recommended YOLO preprocessing guidelines. The image-based procedure was not designed to estimate a comprehensive pavement condition metric such as the PCI. Instead, it was designed to identify visually detectable road surface obstacles—such as potholes, cracks, depressions, patches, and localized discontinuities—that could plausibly interfere with driver behavior and short-term vehicle operation.

**Figure 4:**
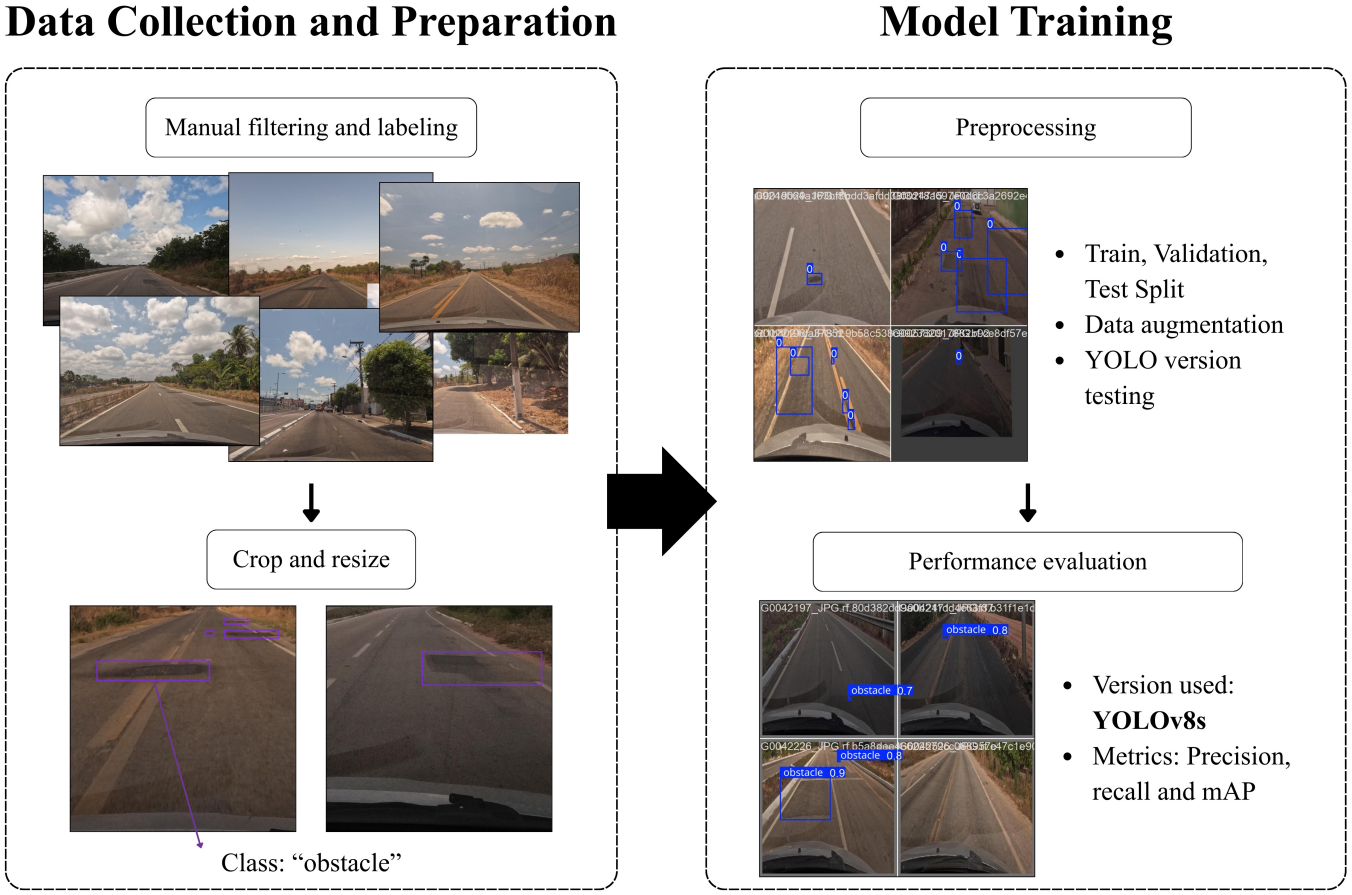
Illustrates the complete workflow adopted for data preparation, preprocessing, and model training.

A subset of images representing diverse pavement conditions was manually annotated using bounding boxes to identify observable surface obstacles, including potholes, cracks, patches, depressions, and localized discontinuities. After filtering and balancing, the final annotated dataset used for training, validation, and testing comprised 1,812 images with 3,158 labeled obstacles, providing adequate representation of both obstacle-present and obstacle-free visual conditions. Data augmentation techniques—such as rotation, flipping, brightness adjustment, and geometric transformations—were applied to enhance model generalization under varying environmental conditions. Figure 4 illustrates the steps used in identifying road obstacles. The annotation protocol therefore prioritized operationally relevant visible obstacles rather than a full engineering characterization of pavement condition severity.

The YOLOv8n model was selected due to its advantageous balance between computational efficiency and detection accuracy, making it suitable for high-volume inference across the entire image dataset. Model training followed standard object-detection procedures using an 80/10/10 train–validation–test split. Performance was evaluated using precision, recall, mAP@50, and mAP@50–95. The metric mAP@50 represents the mean Average Precision at an Intersection-over-Union (IoU) threshold of 0.5, while mAP@50–95 corresponds to the average precision across IoU thresholds from 0.50 to 0.95, providing a more stringent assessment of localization accuracy. The trained model achieved a precision of 0.7722 and a recall of 0.5304, indicating a detection profile oriented toward high-confidence obstacle identification. These values are adequate for the purpose of this study, which uses the detector to derive an image-based indicator of obstacle occurrence for synchronized emission analysis. In methodological terms, these performance metrics refer exclusively to model evaluation on the held-out dataset and are reported here to characterize detector behavior. The Results section subsequently focuses on the application of the trained model to the image sequences corresponding to the five monitored road sections used in the emission analysis. Accordingly, the derived obstacle indicator should be interpreted as an image-based proxy of road surface obstacle occurrence, rather than as an exhaustive inventory of all surface anomalies present along the road.

After validation, the trained model was applied to the image sequences corresponding to the five monitored road sections used in the synchronized emission analysis. Each image was automatically classified as “obstacle-present” when at least one road surface obstacle was detected or “obstacle-free” when no anomalies were identified. A blind-test evaluation using previously unseen images was also conducted to verify generalization performance. For each road segment and driving direction, the total number of analyzed frames and the number of obstacle-present frames were computed. These values were used to calculate the percentage of images containing obstacles, which served as the primary indicator of localized obstacle occurrence in the Results section. More precisely, this percentage should be interpreted as an image-based indicator of localized obstacle occurrence and potential driving disturbance, rather than as a comprehensive index of pavement condition for management purposes.

### 2.5 Data synchronization and pre-processing

PEMS data and YOLO-derived obstacle detections were aligned using GPS timestamps, ensuring precise temporal and spatial matching between emission events and pavement surface conditions. A custom Python routine was developed to segment the roads into contiguous sections classified as “obstacle-free” or “obstacle-present,” based on YOLO detection outputs. Each segment included both forward and backward directions, enabling a bidirectional assessment of surface–emission interactions. Because synchronization was performed at 1-s resolution, the minimum analytical segment corresponded to the distance traveled during 1-s of vehicle operation; therefore, segment length was not fixed a priori in meters and varied according to instantaneous speed. Accordingly, the term “segment” in this study refers to continuous time-synchronized analytical units derived from obstacle occurrence.

Segments containing missing GPS data, signal drift, camera occlusion, or PEMS anomalies were excluded from the analysis. For each valid segment, key variables—including mean and standard deviation of pollutants, instantaneous speed, and acceleration—were computed. This segmentation allowed finer-scale evaluation of how localized pavement obstacles influence dynamic vehicular behavior and pollutant emission rates under real-world conditions. Obstacle-present and obstacle-free segments were therefore compared within the temporal resolution allowed by the synchronized dataset. This approach was intended to support localized emission screening under real driving conditions, while acknowledging that residual confounding from speed variation, traffic interactions, road geometry, and other operational factors may still remain.

### 2.6 Emission metrics and statistical analysis

Emission rates were normalized per unit distance (g·km^−1^ for CO_2_; mg·km^−1^ for CO and NO_x_) following standard on-road testing procedures. For each road segment, descriptive statistics were computed, including mean emission levels, variability indicators, and emission distributions. Speed–acceleration profiles were analyzed to capture transient engine behavior associated with obstacle-induced disturbances. The simultaneous examination of instantaneous speed and pollutant behavior was particularly important because real-world emissions are sensitive to transient operating conditions and short-term engine load changes, which are not fully represented by average-speed approaches alone.

To reduce the influence of observable geometric and operational confounding factors, a post-collection analytical filtering workflow was implemented. A sub-dataset was extracted by excluding vehicle operation data points associated with: (i) horizontal curves and road bends, identified through GPS trajectory curvature and video validation; (ii) a 50-m buffer zone around intersections, roundabouts, and traffic signals; and (iii) visual evidence of direct pedestrian interactions or heavy leading vehicles captured by the forward-facing camera. This procedure retained primarily straight, uninterrupted, free-flow road segments, allowing a sensitivity analysis of emissions in obstacle-present and obstacle-free conditions under reduced external interference. This filtered comparison was intended to evaluate whether the obstacle–emission association persisted after removing the most evident operational confounders, rather than to claim complete causal isolation of pavement effects.

Although this study focused primarily on descriptive and exploratory analysis, the methodological framework supports future inferential modeling. Approaches based on VSP, multivariable regression, telemetric data integration, or machine learning could be incorporated in subsequent stages to estimate adjusted associations and better characterize the mechanisms linking roadway surface obstacles, driving behavior, and emission outcomes. This direction is consistent with recent behavior-based emission frameworks that use VSP and telemetric variables to identify high-emission driving patterns and spatially concentrated emission events under real-world conditions (Muhammad et al., 2024). At the present stage, however, the analysis should be interpreted as high-resolution empirical evidence of association between road obstacle occurrence and pollutant behavior, rather than as a fully controlled inferential design. Future extensions may incorporate VSP-based formulations, multivariable regression, repeated-run designs, or distance-based image acquisition strategies to better separate the contribution of road obstacles from those of speed, traffic, grade, and other contextual variables.

### 2.7 Operational considerations

All field tests were conducted by the same trained driver to minimize variability attributable to driving behavior. A predefined driving protocol was followed, prioritizing smooth operation and adherence to posted speed limits. Data collection was avoided during periods of rainfall, excessive cloud cover, or abnormal traffic conditions to maintain consistency in video quality and vehicle operation. Using the same driver and vehicle throughout the campaign was intended to reduce internal variability in the observed responses, even though this choice necessarily limits the external generalization of the results to other drivers, vehicles, and fleet compositions.

Environmental variables—including ambient temperature, humidity, and wind speed—were logged before each run to document the conditions under which emissions and video data were collected. All procedures complied with local traffic legislation and research safety standards. The multi-instrument setup was secured to avoid interference with visibility or vehicle operation, ensuring that all data acquisition activities were performed safely and reliably. The study design thus prioritized measurement consistency and space–time synchronization across data sources. Nevertheless, because the campaign relied on one instrumented vehicle, the resulting dataset is best understood as an exploratory real-world case study that provides fine-grained empirical evidence rather than fleet-wide or network-wide generalization.

## 3 Results

### 3.1 Obstacle detection performance and overall distribution

A total of five urban road sections were analyzed, each exhibiting distinct pavement conditions and obstacle frequencies. The YOLO-based detection framework identified a heterogeneous distribution of localized road surface obstacles across the network, including potholes, cracks, patches, and depressions. Obstacle occurrence varied substantially among the evaluated roads, ranging from sections where obstacles appeared in more than one-third of the analyzed frames—to well-maintained sections with only isolated irregularities. This spatial heterogeneity reflects the uneven pavement maintenance patterns typical of tropical metropolitan environments and provides a basis for examining whether roadway surface obstacles are associated with changes in vehicular emissions under real-world operating conditions. In the context of this study, these detections should be interpreted, primarily, as localized roadway surface obstacles with potential to interfere with short-term driving behavior.

The overall performance of the obstacle detection model is summarized in Table 1. Precision values ranged from 0.7878 to 0.9761, indicating that the model maintained a high-confidence detection profile across the monitored road sections. Recall values showed greater variation, ranging from 0.3846 to 0.6585, which is expected in image-based detection tasks involving irregularly shaped, visually heterogeneous road obstacles. The mAP@50 values (0.5771–0.7597) demonstrated satisfactory localization performance at the standard IoU threshold of 0.5, whereas the more stringent mAP@50–95 values (0.3534–0.4866) reflected the increased difficulty of precisely bounding irregularly shaped obstacles under diverse lighting and pavement textures. Together, these metrics confirm the model's suitability for generating reliable image-based obstacle indicators for use in subsequent analyses. Because model calibration and validation were already described in Section 2.4, the present subsection emphasizes the practical behavior of the trained detector when applied to the five monitored corridors. The observed precision–recall combination supports the use of the detector as a practical screening tool for identifying the main categories of visually detectable road surface obstacles considered in this study.

**Table 1:** Performance of YOLO-based obstacle detection, analyzed segment length and obstacle occurrence across the five monitored road sections.

Road	Segment length (km)	Precision	Recall	mAP@50	mAP@50-95	Frames	Obstacles	Obstacle percentage (%)
Road 1	2.8770	0.8284	0.5953	0.7236	0.4866	466	146	31.33
Road 2	2.5440	0.8437	0.6585	0.7597	0.4489	368	82	22.23
Road 3	4.0100	0.7878	0.4251	0.6377	0.3891	553	98	24.96
Road 4	3.1820	0.8128	0.4933	0.6303	0.3802	648	75	11.57
Road 5	4.4420	0.9761	0.3846	0.5771	0.3534	275	13	4.73

The percentage of obstacle-present images varied widely among the five road sections, serving as an objective and interpretable indicator of localized obstacle occurrence and potential driving disturbance. Road 1 displayed the highest proportion of obstacle-present frames (31.33%), followed closely by Roads 3 (24.96%) and 2 (22.23%). These values indicate recurrent surface irregularities consistent with visible signs of localized surface obstacles, such as cracking and rutting, observed during field inspections. Conversely, Road 5 exhibited the lowest obstacle percentage (4.73%), confirming its status as the section with the lowest occurrence of visually detected road obstacles within the dataset. Road 4 presented an intermediate obstacle share (11.57%), reflecting moderate but non-negligible obstacle occurrence. Given the heterogeneous distribution of obstacles detected by YOLO, the next step was to examine how these conditions are spatially associated with the distribution of pollutant emissions.

### 3.2 Spatial distribution of pollutant emissions along the road segments

**Figure 5:**
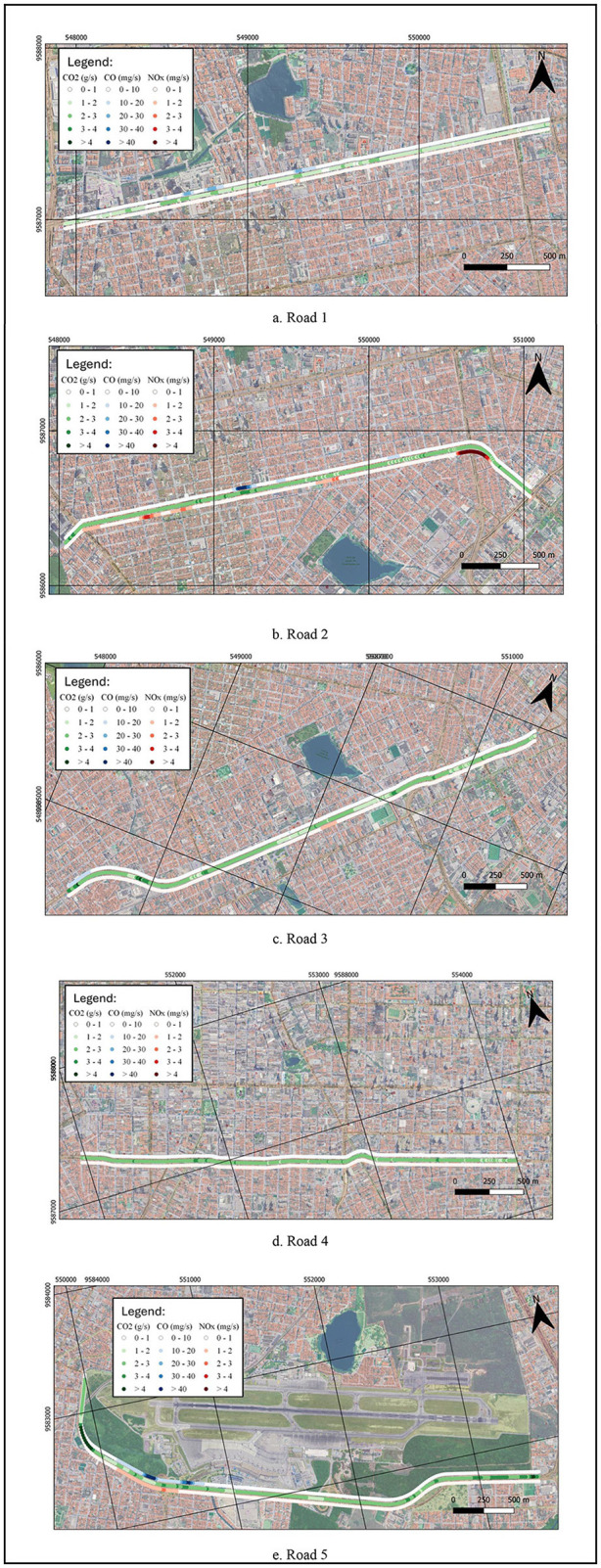
Spatial distribution of CO_2_, CO, and NO_x_ emissions across the five urban road sections analyzed in this study. **(a)** Road 1; **(b)** Road 2; **(c)** Road 3; **(d)** Road 4; **(e)** Road 5.

The spatial distribution of CO_2_, CO, and NO_x_ emissions for the five evaluated road sections is shown in Figure 5a– e. Each marker represents a one-second emission measurement georeferenced along the vehicle trajectory, classified into intensity ranges for the three pollutants. This visualization enables a detailed assessment of localized emission patterns and their spatial variability within each corridor.

Overall, the mapped emissions reveal heterogeneous patterns across all road segments. CO_2_ displays the broadest spatial continuity, forming gradients along longer stretches that reflect variations in engine load associated with changes in speed and acceleration. CO exhibits more discrete and isolated hotspots, generally corresponding to rapid deceleration events or short-duration transients in combustion efficiency. NO_x_ presents an intermediate behavior, with moderate background values punctuated by short but well-defined high-intensity segments, particularly in areas of increased vehicle demand such as curves and intersection approaches. This pattern is consistent with the broader on-road emissions literature, which shows that transient operating conditions and route characteristics can substantially influence short-duration pollutant peaks (Zhai et al., 2008; Weiss et al., 2011). The spatial concentration of emission peaks is also consistent with recent behavior-based emission frameworks that emphasize the role of driving dynamics and high-emission zones in real-world urban operation (Muhammad et al., 2024).

When evaluating the filtered sub-dataset composed of straight, free-flow segments outside curves, intersection buffers, and visually identified pedestrian or heavy-traffic interference, the association between localized roadway surface obstacles and pollutant emissions remained observable. In this restricted subset, obstacle-present observations showed average increases of 26% for CO_2_, 31% for NOx, and 42% for CO compared with obstacle-free observations under comparable operating conditions. These increases were lower than the gross differences observed in the unfiltered dataset, indicating that part of the corridor-level emission contrast was amplified by operational factors such as stop-and-go movements, intersection approaches, and cornering maneuvers. Nevertheless, the persistence of higher emissions in the filtered subset suggests that localized roadway surface obstacles may contribute to short-term emission variability even when major observable confounders are reduced. This residual association is plausibly related to micro-accelerations, steering corrections, and instantaneous engine-load adjustments induced by driver responses to potholes, depressions, and other localized surface discontinuities.

In Road 1 (Figure 5-a), emissions of all pollutants are relatively dispersed, with CO_2_ showing the largest spatial extent of elevated values. Small clusters of CO and NO_x_ appear near segments where the road narrows or where turning maneuvers occur. In Road 2 (Figure 5-b), emissions are more spatially concentrated, especially NO_x_ peaks aligned with geometric deflection points and downstream of intersections. CO hotspots also appear adjacent to abrupt speed changes. Road 3 (Figure 5-c) shows the most uniform CO_2_ profile among all segments, although localized NO_x_ spikes are visible near driveway entrances and zones of higher pedestrian activity. In Road 4 (Figure 5-d), emissions remain mostly stable along the central portion of the segment, with only a few scattered hotspots of CO and NO_x_ occurring near minor intersections. Finally, Road 5 (Figure 5-e) exhibits limited emission variability due to its smoother alignment and fewer operational disturbances, although elevated CO and NO_x_ values appear near the terminal curve, where speed reductions were more pronounced.

Although Road 5 has the greatest segment length among the monitored sections, it presented the lowest number of analyzed frames. This pattern is consistent with the temporal sampling strategy adopted in this study: because images were extracted at fixed 1-s intervals, higher average travel speeds increase the spatial distance between consecutive analyzed frames. For this reason, the spatial interpretation of obstacle occurrence along Road 5 should be based on the joint reading of Figure 5 and the segment-length information added to Table 1, rather than on frame count alone. More generally, the spatial results suggest that pollutant hotspots are not uniformly distributed along each corridor, reinforcing the importance of localized roadway disturbances, geometry, and short-term operational changes in shaping emission behavior.

### 3.3 Pollutant emission profiles across road sections

Table 2 summarizes the mean emission rates of CO_2_ (g/s), CO (mg/s), and NO_x_ (mg/s), as well as distance-normalized values (g/km and mg/km) for the five urban road sections. Substantial variability was observed across pollutants and road segments, reflecting the diverse driving dynamics and road surface and operational conditions encountered during the field experiments. These values should be interpreted as corridor-level descriptive summaries, while recognizing that each road section includes localized spatial variability in geometry, speed profile, and obstacle occurrence.

**Table 2:** Emission statistics (mean, standard deviation, and distance-normalized rates) for CO_2_, CO, and NO_x_ along Roads 1–5.

Mean		Road 1	Road 2	Road 3	Road 4	Road 5
**CO**_**2**_ **(g s**^**−1**^**)**	**Mean**	1.5037	2.3612	2.4300	2.3443	2.6194
	**stdDev**	**0.4558**	**0.3736**	**0.3909**	**0.2595**	**0.5963**
**CO (mg s** ^**−1**^ **)**	**Mean**	0.2500	3.1046	1.1150	0.0014	3.0114
	**stdDev**	**0.6266**	**6.6628**	**5.6024**	**0.0364**	**9.3975**
**NO**_**x**_ **(mg s**^**−1**^**)**	**Mean**	0.0055	0.6327	0.1113	0.0923	0.1609
	**stdDev**	**0.0122**	**1.2218**	**0.0557**	**0.0497**	**0.3654**
**CO**_**2**_ **(g km**^**−1**^**)**		244.445	374.256	370.342	478.242	157.145
**CO (mg km** ^**−1**^ **)**		40.640	491.960	169.890	0.291	180.599
**NO**_**x**_ **(mg km**^**−1**^**)**		0.886	100.266	16.951	18.333	9.649

CO_2_ exhibited the highest magnitude among the measured pollutants, with mean instantaneous rates ranging from 1.50 to 2.62 g/s across the five roads. When normalized by distance traveled, Road 4 recorded the highest CO_2_ emission factor (478.2 g/km), followed by Roads 2 and 3 (374.2 and 370.3 g/km, respectively). Road 5 showed the lowest CO_2_ intensity (157.1 g/km), consistent with its smoother surface and lower obstacle percentage reported earlier. These results suggest that sustained acceleration events, transient load increases, and local operating conditions may contribute to elevated CO_2_ release along specific corridors. This interpretation is consistent with previous PEMS-based evidence indicating that route characteristics and transient operating modes materially affect distance-specific emissions under real driving conditions (Weiss et al., 2011). It is also consistent with studies showing that pavement roughness can influence emissions both directly and indirectly through driver speed choice and operating behavior (Chen et al., 2024).

CO presented more pronounced differences among road sections. Road 2 exhibited the highest mean CO rate (3.1046 mg/s), closely followed by Road 5 (3.0114 mg/s), whereas Road 4 recorded the lowest value (0.0014 mg/s). The elevated CO levels observed in Roads 2 and 5 correspond to segments identified earlier as containing localized hotspots of transient driving, such as sharp curves or speed reductions. These conditions typically may be associated with short-term combustion inefficiencies and higher CO emissions. At the same time, the contrast between instantaneous and distance-normalized values suggests that road-level averages are shaped not only by obstacle occurrence, but also by the interaction between speed profile, travel time, and local operating mode along each corridor.

NO_x_ emissions displayed the strongest contrast across road sections. Road 2, in particular, exhibited a mean NO_x_ rate of 0.6327 mg/s, resulting in a distance-normalized emission factor of 100.266 mg/km—the highest among all segments. In contrast, Road 1 and Road 5 presented markedly lower NO_x_ values, emitting approximately 99 and 90% less, respectively, than Road 2 (0.886 and 9.649 mg/km vs. 100.266 mg/km). This pattern aligns with the presence of abrupt geometric deflections and obstacle clusters along Road 2, which likely intensified engine load and combustion temperature, thereby promoting NO_x_ formation. Such sensitivity of NO_x_ to operating mode and route severity is also compatible with previous on-road findings showing that NO_x_ emissions may vary substantially with load pattern, speed, and route characteristics (Weiss et al., 2011). However, because speed, geometry, and obstacle occurrence co-vary under real-world conditions, these contrasts should be interpreted as descriptive evidence of association rather than as isolated effects of roadway surface obstacles alone.

Overall, the emission profiles reveal a consistent tendency: road sections with higher obstacle density and more irregular driving dynamics exhibit considerably higher pollutant release, particularly for CO_2_ and NO_x_. Roads 2 and 3, which contained some of the highest percentages of obstacle-present frames, also exhibited the most elevated emission factors. Conversely, Road 5—characterized by the lowest obstacle prevalence—displayed the lowest emission intensities for all three pollutants. The results are consistent with the hypothesis that localized roadway surface obstacles may disturb short-term vehicle operation and contribute to higher pollutant emissions under real urban driving conditions. Therefore, the results support an empirical association between behaviorally relevant roadway obstacles, transient vehicle operation, and pollutant variability, while remaining consistent with the exploratory design of the study.

## 4 Discussion

The results obtained in this study demonstrate a relationship between pavement surface obstacles and real-world vehicular emissions. Across the five urban road sections analyzed, obstacle-dense segments exhibited up to threefold higher CO_2_ emissions, over 99% higher CO emissions, and up to 90% higher NO_x_ emissions compared with well-maintained segments. These magnitudes reinforce the hypothesis that localized roadways surface obstacles may alter vehicle dynamics and trigger transient engine operation, ultimately leading to less efficient combustion. This behavior is consistent with previous evidence showing that acceleration–deceleration cycles and unstable driving patterns substantially increase real-world emissions, as reported by Shahare et al. (2017). It is also consistent with recent evidence indicating that pavement roughness may affect emissions not only through rolling resistance, but also indirectly by influencing drivers' speed choice and short-term operating behavior (Chen et al., 2024). The present findings should be interpreted as strong empirical evidence of association between localized roadway surface obstacles and higher pollutant levels under real urban driving conditions, rather than as fully isolated effect estimates.

The observed variation in CO_2_ emissions across the different road sections aligns with global assessments showing that infrastructure quality influences fuel consumption and carbon output. Fuller et al. (2022) highlight that urban air pollution is strongly shaped by a combination of vehicle activity and environmental context, with infrastructure conditions acting as an important modifier of emission intensity and air quality. Although Fuller et al. (2022) focuses on exposure and health, their findings match the broader view that emissions are highly sensitive to micro-scale features of the urban environment. This broader interpretation is also supported by studies linking pavement smoothness and roughness to operational fuel consumption. Using connected vehicle data in a before–after design, Llopis-Castelló et al. (2026) reported that improved pavement smoothness was associated with reductions of approximately 10% in both fuel consumption and CO_2_ emissions. Similarly, Chen et al. (2024) proposed a pavement-performance-based carbon-emission model showing that roughness can influence vehicle carbon emissions through both direct physical mechanisms and indirect behavioral responses related to speed selection. This is particularly relevant to the present study because the analyzed obstacles are not interpreted merely as pavement distresses, but as surface features with potential to alter the driving pattern.

The elevated CO_2_ and NO_x_ levels observed in deteriorated segments of Road 2 align with the factors identified by Fontaras et al. (2017), who demonstrated that real-world energy demand is substantially increased by environmental and road-related conditions not represented in laboratory certification tests. Their review highlights that road roughness, rolling resistance, transient driving, and surface anomalies can significantly elevate fuel consumption and tailpipe emissions, reinforcing that road surface irregularities may act as relevant contributors to real-world emission variability. The present results are compatible with that interpretation, particularly because the highest pollutant levels were not uniformly distributed along the monitored corridors, but instead tended to cluster in locations where roadway geometry, speed adjustment, and localized surface obstacles were more evident. This suggests that roadway conditions act together with operational context, rather than in isolation, to shape instantaneous emission behavior. Therefore, the observed spatial patterns should be understood as the combined result of obstacle occurrence, local geometry, speed variation, and short-term driver response, rather than as the isolated effect of pavement condition alone.

More specifically, the results complement experimental findings Oliveira et al. (2025), which revealed measurable differences in NO_x_ emissions between asphalt and concrete pavements even under constant-speed conditions. While that paper isolates pavement type, the current study extends this understanding by demonstrating that localized surface obstacles may exert an additional influence on emissions, particularly because they induce transient dynamics that constant-speed tests cannot capture. In this sense, the contribution of the present study lies less in comparing broad pavement classes and more in showing that micro-scale roadway disturbances observable from onboard imagery may be associated with relevant short-term emission changes under everyday urban circulation. This distinction is important because the objective here is not to derive a complete pavement-condition score, but to identify operationally relevant roadway surface obstacles that can interfere with driver behavior and vehicle response. This framing is consistent with the behavioral interpretation proposed by Chen et al. (2024), in which pavement-related effects on emissions are mediated in part by driver speed choice and operating decisions.

The integration of PEMS with YOLO-based obstacle detection proved effective in linking emissions to precise spatial features. This methodological coherence is supported by Weiss et al. (2011), who demonstrated that PEMS can capture emission peaks linked to dynamic driving events that are otherwise concealed in laboratory protocols (Zhai et al., 2008). The present study advances this logic by synchronizing second-by-second emission measurements with image-based obstacle occurrence, thereby enabling localized screening of pollutant behavior in relation to visually detectable roadway disturbances. Rather than functioning as a substitute for conventional pavement inspection metrics, the YOLO-based procedure operated here as a screening tool for detecting roadway surface obstacles with potential operational relevance. This combined approach is particularly valuable in urban settings, where short-duration events often dominate real-world emissions and where average corridor-level indicators may conceal important micro-scale variability. Recent behavior-based and telemetric emission frameworks similarly emphasize that real-world emissions are strongly influenced by instantaneous driving behavior, VSP-related operating conditions, and spatially concentrated high-emission zones (Muhammad et al., 2024). Thus, the integration of PEMS and image-based detection adopted here is consistent with a broader shift toward high-resolution, spatial–temporal approaches for interpreting urban emission variability.

The current results suggest that infrastructure-induced dynamics play a measurable role in emissions and should be considered in future emission inventories. More specifically, the results suggest that roadway surface condition—when expressed in terms of localized obstacles capable of disturbing short-term driving behavior—may represent a relevant explanatory layer in real-world emission assessment. This interpretation is consistent with the recent review by Nitoiu et al. (2025), which emphasizes that road and traffic characteristics jointly shape urban driving cycles, fuel consumption, and pollutant emissions. Accordingly, future emission inventories and predictive models may benefit from incorporating not only conventional traffic and fleet variables, but also infrastructure-related descriptors that reflect the operational consequences of localized roadway irregularities. This point is reinforced by Muhammad et al. (2024), who showed that data-driven frameworks based on driving behavior and VSP can help identify high-emission patterns and spatial hotspots under real-world conditions. In future applications, obstacle occurrence indicators such as those proposed in this study could be combined with VSP, speed–acceleration profiles, road grade, and traffic variables to improve emission modeling and scenario assessment.

Moreover, in a climate context, Molina et al. (2019) underscore that reductions in urban NO_x_ and CO emissions produce meaningful improvements in air quality. Given that obstacle-dense segments in the present study produced up to three times more pollutant emissions than well-maintained ones, pavement rehabilitation in Brazilian tropical cities may offer a potential opportunity for integrated infrastructure, air-quality, and climate co-benefits. At the same time, this implication should be interpreted with caution, because the present dataset was obtained from one instrumented vehicle operating under naturally varying geometric and traffic conditions. Even so, the magnitude and consistency of the observed contrasts suggest that roadway maintenance may yield environmental co-benefits beyond ride quality and infrastructure performance, particularly in urban corridors where localized surface obstacles are frequent and operational disturbances are recurrent. From a policy perspective, these results support the view that pavement maintenance should also be discussed as part of integrated urban air-quality and climate strategies, especially in tropical metropolitan environments where climatic exposure may accelerate surface deterioration and amplify its operational consequences. However, future studies should include repeated runs, fixed-distance image acquisition, broader vehicle fleets, and multivariable modeling to better separate the contribution of road obstacles from the effects of speed, traffic flow, geometry, and environmental conditions.

### 4.1 Confounding biases and policy relevance

Evaluating vehicle emissions under real-world urban conditions introduces unavoidable confounding factors that may affect the interpretation of the observed association between roadway surface obstacles and pollutant emissions. Table 3 systematically outlines the identified confounding factors, their hypothesized direction of bias, estimated magnitude relative to the pavement structural signal, and implications for policy translation.

**Table 3:** Methodological confounding factors, bias quantification, and interpretation.

Confounding factor	Potential bias direction	Estimated magnitude and significance	Impact on interpretation and policy
**Road geometry (curves/bends)**	Overestimation of pavement impact (if irregularities cluster on bends)	Moderate-high. Bends increase lateral engine load and emissions independently.	Overestimates the structural pavement signal if not filtered; requires geometry-aware infrastructure prioritization.
**Intersection/traffic flow proximity**	Overestimation of pavement impact (due to deceleration/acceleration cycles)	High. Stop-and-go cycles generate transient emission peaks that easily dwarf baseline pavement rolling resistance.	Part of the emission peak is operational, not structural. Infrastructure funding should prioritize fixing pavements near intersections to mitigate compound emissions.
**YOLO detector recall limits (false negatives)**	Underestimation of pavement impact	Low-moderate. missed obstacles (recall: ~0.53) categorize a truly degraded section as “obstacle-free”.	Biases the “obstacle-free” baseline upward. The reported emission reductions for well-maintained segments are therefore conservative (underestimated).
**Single test vehicle/driver setup**	Neutral (internal validity)/unknown (external generalization)	Variable. minimizes behavior variance but omits fleet-wide suspension dynamics and weight configurations.	High internal robustness for establishing the association mechanism, but limits immediate macroscopic emission factor modeling for heterogeneous fleets.

As indicated in Table 3, some operational factors, particularly intersection proximity and stop-and-go movements, are likely to overestimate the apparent contribution of roadway surface obstacles by introducing emission peaks related to acceleration and deceleration cycles. Conversely, limitations in obstacle detection recall may underestimate the obstacle effect by classifying some obstacle-present observations as obstacle-free, thereby increasing the emission baseline of the comparison group. The filtered analysis therefore provides a more conservative interpretation of the obstacle–emission relationship, although it does not eliminate all sources of residual confounding. From a policy perspective, the results should not be interpreted as definitive emission factors for pavement maintenance interventions, but as evidence that roadway surface conditions may be relevant enough to be considered in integrated infrastructure, air-quality, and climate-mitigation planning.

## 5 Conclusion and future work

This study provides high-resolution empirical evidence that localized roadway surface obstacles are associated with changes in vehicular emissions under real-world urban driving conditions. By integrating second-by-second PEMS measurements with YOLO-based image detection, the analysis showed that obstacle-dense road sections presented higher CO_2_, CO, and NOx emissions than better-maintained sections. In the full dataset, the highest emission factors reached 478.2 g/km for CO_2_, 491.96 mg/km for CO, and 100.266 mg/km for NOx. Importantly, after filtering out major observable confounders—including curves, intersection buffers, and visible pedestrian or heavy-traffic interference—the obstacle–emission association remained observable, with obstacle-present observations showing average increases of 26% for CO_2_, 31% for NOx, and 42% for CO relative to obstacle-free observations. These results suggest that localized roadway surface obstacles may contribute to short-term emission variability even under reduced external interference.

The main contribution of this study lies in reframing pavement-related emission analysis around behaviorally relevant roadway obstacles rather than conventional pavement condition indices. The image-based indicator used here was not intended to replace PCI or network-level pavement management metrics. Instead, it was designed to identify visible surface discontinuities—such as potholes, cracks, depressions, and patches—that may induce steering corrections, braking, speed adjustments, micro-accelerations, and instantaneous engine-load changes. This interpretation is consistent with previous evidence showing that pavement roughness and surface irregularities can affect emissions both directly through rolling resistance and indirectly through driver speed choice and vehicle operating behavior (Fontaras et al., 2017; Chen et al., 2024; Llopis-Castelló et al., 2026).

From a policy perspective, the findings indicate that roadway maintenance may generate environmental co-benefits beyond ride quality, safety, and asset preservation. In urban corridors where localized obstacles are frequent, maintenance actions may reduce operational disturbances associated with higher pollutant release and may therefore contribute to integrated transport, air-quality, and climate-mitigation strategies. However, the results should not be interpreted as definitive emission factors for pavement rehabilitation interventions. Rather, they provide evidence that roadway surface conditions are relevant enough to be considered in urban emission assessment, especially when combined with traffic, fleet, and operational variables.

Several limitations should be considered when interpreting these results. First, the study was based on five urban corridors in one tropical city, which limits spatial generalization. Second, the use of a single light-duty vehicle and one trained driver improved internal consistency but does not represent the variability of broader vehicle fleets, including buses, motorcycles, heavy-duty vehicles, or different powertrain technologies. Third, image extraction at 1 Hz supported temporal synchronization with PEMS data but did not provide fixed-distance pavement sampling. Fourth, although the filtered analysis reduced major observable confounders, residual effects from speed variation, traffic flow, road grade, geometry, and surrounding driving context may still remain. Finally, the YOLO detector should be interpreted as an image-based proxy of obstacle occurrence, not as an exhaustive inventory of all surface anomalies.

Future investigations should address these limitations through four complementary directions. First, the spatial and temporal coverage of field campaigns should be expanded to include more corridors, road classes, traffic regimes, seasonal conditions, and land-use contexts. This would improve statistical representativeness and clarify whether the observed obstacle–emission association is transferable across different urban environments. Second, broader vehicle and driver samples should be incorporated, including passenger cars, motorcycles, buses, and freight vehicles, because suspension response, vehicle mass, fuel type, and driving style may mediate how surface obstacles affect emissions. Third, future studies should integrate richer vehicle-dynamics variables, such as engine load, torque, road grade, acceleration, vertical vibration, and real-time vehicle specific power (VSP), enabling multivariable regression, structural equation modeling, or machine-learning approaches to separate roadway effects from traffic and behavioral effects more explicitly (Muhammad et al., 2024). Finally, obstacle characterization should be improved by distinguishing type, severity, geometry, spatial concentration, and recurrence, while distance-based image acquisition and before–after monitoring of maintenance interventions should be used to estimate potential emission reductions associated with roadway surface improvements. Together, these advances would strengthen the robustness, transferability, and policy relevance of the proposed framework.

## Data Availability

The raw data supporting the conclusions of this article will be made available by the authors, without undue reservation.
